# Predicting the functional consequences of cancer-associated amino acid substitutions

**DOI:** 10.1093/bioinformatics/btt182

**Published:** 2013-05-17

**Authors:** Hashem A. Shihab, Julian Gough, David N. Cooper, Ian N. M. Day, Tom R. Gaunt

**Affiliations:** ^1^Bristol Centre for Systems Biomedicine and MRC CAiTE Centre, School of Social and Community Medicine, University of Bristol, Bristol BS8 2BN, ^2^Department of Computer Science, University of Bristol, The Merchant Venturers Building, Bristol BS8 1UB and ^3^Institute of Medical Genetics, School of Medicine, Cardiff University, Cardiff CF14 4XN, UK

## Abstract

**Motivation:** The number of missense mutations being identified in cancer genomes has greatly increased as a consequence of technological advances and the reduced cost of whole-genome/whole-exome sequencing methods. However, a high proportion of the amino acid substitutions detected in cancer genomes have little or no effect on tumour progression (passenger mutations). Therefore, accurate automated methods capable of discriminating between driver (cancer-promoting) and passenger mutations are becoming increasingly important. In our previous work, we developed the Functional Analysis through Hidden Markov Models (FATHMM) software and, using a model weighted for inherited disease mutations, observed improved performances over alternative computational prediction algorithms. Here, we describe an adaptation of our original algorithm that incorporates a cancer-specific model to potentiate the functional analysis of driver mutations.

**Results:** The performance of our algorithm was evaluated using two separate benchmarks. In our analysis, we observed improved performances when distinguishing between driver mutations and other germ line variants (both disease-causing and putatively neutral mutations). In addition, when discriminating between somatic driver and passenger mutations, we observed performances comparable with the leading computational prediction algorithms: SPF-Cancer and TransFIC.

**Availability and implementation:** A web-based implementation of our cancer-specific model, including a downloadable stand-alone package, is available at http://fathmm.biocompute.org.uk.

**Contact:** fathmm@biocompute.org.uk

**Supplementary information:** Supplementary data are available at *Bioinformatics* online.

## 1 INTRODUCTION

Human cancers are characterized by the accumulation of somatic mutations, e.g. gross insertions and deletions, as well as the more subtle single base pair substitutions (Iengar, 2012), some of which confer a growth advantage on the tumour cells (Hanahan and Weinberg, 2011). The Catalogue of Somatic Mutations in Cancer (COSMIC) (Bamford *et al.*, 2004) is an online repository of somatic mutation data, which includes amino acid substitutions (AASs). The identification of cancer-promoting AASs (driver mutations) promises to lead to a better understanding of the molecular mechanisms underlying the disease, as well as providing potential diagnostic and therapeutic markers (Furney *et al.*, 2006). However, this remains a major challenge, as the majority of AASs detected in cancer genomes do not contribute to carcinogenesis; rather, these ‘passenger mutations’ are a consequence of tumorigenesis rather than a cause (Greenman *et al.*, 2007). Therefore, accurate automated computational prediction algorithms capable of distinguishing between driver and passenger mutations are of paramount importance.

A review by Thusberg *et al.* (2011) describes the performance of several computational prediction algorithms (Adzhubei *et al.*, 2010; Bao *et al.*, 2005; Bromberg and Rost, 2007; Calabrese *et al.*, 2009; Capriotti *et al.*, 2006; Li *et al.*, 2009; Ng and Henikoff, 2001; Mort *et al.*, 2010; Ramensky *et al.*, 2002; Thomas *et al.*, 2003) using a ‘gold standard’ validation benchmark (Sasidharan Nair and Vihinen, 2013). In our previous work, we developed the Functional Analysis through Hidden Markov Models (FATHMM) algorithm and, using a model weighted for inherited disease mutations, observed improved performance accuracies over alternative computational prediction methods using the same benchmark (Shihab *et al.*, 2013). However, the value of traditional computational prediction algorithms in cancer genomics remains unclear (Kaminker *et al.*, 2007a). For example, the shared characteristics between driver and other disease-causing mutations allow for a significant proportion of cancer-associated mutations to be identified (high-sensitivity/true positive rate); however, these methods are incapable of reliably distinguishing between driver and other disease-causing mutations. Furthermore, with respect to carcinogenesis, a large proportion of passenger mutations are still misclassified as having a role in tumour progression (low-specificity/true negative rate). As a result, several cancer-specific computational prediction algorithms capable of distinguishing between driver mutations and other germ line variants (both disease-causing and putatively neutral mutations) and/or capable of discriminating between somatic driver and passenger mutations have been developed (Carter *et al.*, 2009; Gonzalez-Perez *et al.*, 2012; Kaminker *et al.*, 2007b; Reva *et al.*, 2011).

In this work, we describe an adaptation to our original algorithm, which amalgamates sequence conservation within hidden Markov models (HMMs), representing the alignment of homologous sequences and conserved protein domains, with ‘pathogenicity weights’, representing the overall tolerance of the corresponding model to mutations (Shihab *et al.*, 2013), to potentiate the functional analysis of driver mutations. Using a model weighted for cancer-associated mutations, we observe performance accuracies, which outperform alternative computational prediction algorithms (Adzhubei *et al.*, 2010; Capriotti and Altman, 2011; Ng and Henikoff, 2001; Reva *et al.*, 2011) when distinguishing between driver and other germ line mutations (both disease-causing and neutral polymorphisms). Furthermore, when discriminating between driver and passenger mutations (somatic), we observe performance accuracies comparable with other state-of-the-art computational prediction algorithms (Capriotti and Altman, 2011; Carter *et al.*, 2009; Gonzalez-Perez *et al.*, 2012). A web-based implementation of our algorithm, including a high-throughput batch submission facility and a downloadable stand-alone package, is available at http://fathmm.biocompute.org.uk.

## 2 METHODS

### 2.1 The mutation datasets

The mutation datasets used in this study were collected and assembled as follows: first, cancer-associated mutations (germ line and somatic) from the CanProVar database (Li *et al.*, 2010) (CanProVar—Version 54; http://bioinfo.vanderbilt.edu/canprovar) and putative neutral polymorphisms from the UniProt database (Apweiler *et al.*, 2004) (UniProt—November 2011; http://www.uniprot.org/docs/humsavar) were downloaded and used to calculate our ‘cancer-specific pathogenicity weights’. Next, we obtained three mutation datasets (Capriotti and Altman, 2011) and performed an independent benchmark comparing the performance of our algorithm with the performance of five alternative computational prediction algorithms (Adzhubei *et al.*, 2010; Capriotti and Altman, 2011; Ng and Henikoff, 2001; Reva *et al.*, 2011). Finally, we obtained a published benchmark consisting of nine mutation datasets (Gonzalez-Perez *et al.*, 2012) and compared the performance of our algorithm with the performance of four alternative computational prediction algorithms (Adzhubei *et al.*, 2010; Gonzalez-Perez *et al.*, 2012; Ng and Henikoff, 2001; Reva *et al.*, 2011). The composition of these datasets is summarized in Table 1, and the overlap between our training and benchmarking datasets is illustrated in Supplementary Table S1.

**Table 1.** Summary of mutation datasets used in this study

Dataset	Positives	Negatives	Description
Training datasets			
CanProVar	12 720	—	A collection of cancer-associated mutations used to calculate our pathogenicity weights
UniProt	—	36 928	A collection of putative neutral polymorphisms used to calculate our pathogenicity weights
Capriotti and Altman benchmark			
CNO	3163	3163	Comprising driver mutations used to train the CHASM algorithm and neutral polymorphisms
CND	3163	3163	Comprising driver mutations used to train the CHASM algorithm and other germ line mutations (both disease-causing and neutral polymorphisms)
Synthetic	3163	3163	Comprising driver and passenger mutations (somatic) used to train the CHASM algorithm
Gonzalez-Perez *et al.* benchmark			
COSMIC 2 + 1	3978	39 850	Comprising COSMIC mutations occurring in 2+ samples and COSMIC mutations occurring in one sample
COSMIC 5 + 1	1631	39 850	Comprising COSMIC mutations occurring in 5+ samples and COSMIC mutations occurring in one sample
COSMIC 2/POL	3978	8040	Comprising COSMIC mutations occurring in 2+ samples and neutral polymorphisms
COSMIC 5/POL	1631	8040	Comprising COSMIC mutations occurring in 5+ samples and neutral polymorphisms
COSMIC D/O	2151	41 664	Comprising driver mutations used to train the CHASM algorithm and COSMIC mutations not in the positive subset
COSMIC D/POL	2151	8040	Comprising driver mutations used to train the CHASM algorithm and neutral polymorphisms
COSMIC CGC/NONCGC	4865	34 827	Comprising COSMIC mutations falling within genes defined in the CGC and COSMIC mutations falling within genes outside the CGC
WG 2/1	790	24 079	Comprising somatic mutations occurring in 2+ samples and somatic mutations occurring in one sample
WG CGC/NONCGC	1302	22 983	Comprising somatic mutations falling within genes defined in the CGC and somatic mutations falling within genes outside the CGC

CGC, Cancer Gene Census (Futreal *et al.*, 2004).

### 2.2 Scoring cancer-associated amino acid substitutions

Following the procedure described in Shihab *et al.* (2013): protein domain annotations from the SUPERFAMILY (Gough *et al.*, 2001) (version 1.75) and Pfam (Sonnhammer *et al.*, 1997) (Pfam-A and Pfam-B; version 26.0) databases are made. Next, the corresponding HMMs are extracted if the mutation maps onto a match state within the model, and the domain assignment is deemed to be significant (e-value ≤0.01). Where multiple HMMs are extracted, then the model with the largest information gain (as measured by the Kullback–Leibler divergence (Kullback and Leibler, 1951) from the SwissProt/TrEMBL amino acid composition) is used. Finally, we interrogate the amino acid probabilities within the model and assume that a reduction in the amino acid probabilities (when comparing the wild-type with the mutant residue) indicates a potential negative impact on protein function. Finally, the predicted magnitude of effect is weighted using cancer-specific pathogenicity weights (Supplementary Methods):
(1)
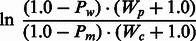



Here, *P_w_* and *P_m_* represent the underlying probabilities for the wild-type and mutant amino acid residues, respectively, and the pathogenicity weights, *W_c_* and *W_p_*, represent the relative frequencies of cancer-associated (CanProVar) and putative neutral polymorphisms (UniProt) mapping onto the relevant HMMs, respectively. A pseudo-count of 1.0 is incremented to our pathogenicity weights to avoid zero divisible terms.

### 2.3 Extending our algorithm to mutations falling outside conserved protein domains

The main disadvantage of our original algorithm was confining coverage (via the weighting scheme used) to protein missense variants falling within conserved protein domains. To increase coverage, we have developed an extension to the aforementioned data for predicting the functional effects of AASs falling outside conserved protein domains. In brief, *ab initio* HMMs, representing the alignment of homologous sequences within the SwissProt/TrEMBL database (Apweiler *et al.*, 2004), are constructed using the *JackHMMER* component of HMMER3 (Eddy, 2009) (one iteration with the optional—*hand* parameter applied). The predicted magnitude of effect is then calculated as in Equation (1); however, these models are weighted with the relative frequencies of cancer-associated (CanProVar) and putative neutral polymorphisms (UniProt) mapping onto the top scoring sequence(s), and their homologous domain(s), being used to construct the model (Supplementary Methods).

### 2.4 Performance evaluation

As recommended in Vihinen (2012), the performance of our method was assessed using the following six parameters [Equations (2–7)]:
(2)
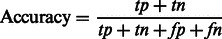

(3)
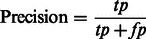

(4)
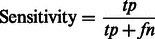

(5)
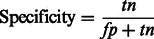

(6)
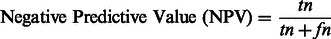

(7)
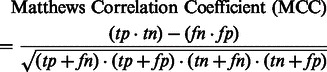



In the aforementioned data, *tp* and *fp* refer to the number of true positives and false positives reported and *tn* and *fn* denote the number of true negatives and false negatives reported.

## 3 RESULTS

### 3.1 A cancer-specific prediction threshold

**Fig. 1.**
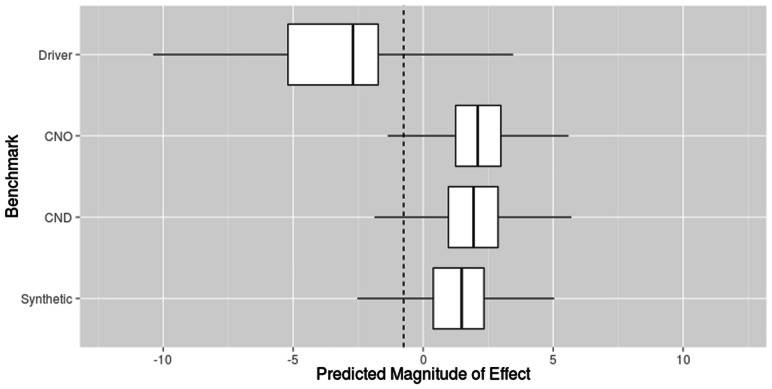
The distribution of the predicted magnitude of effect for all driver mutations against all non–cancer-associated (germ line and somatic) mutations in the Capriotti and Altman (2011) benchmark. Here, the dashed line represents our prediction threshold of −0.75 at which the specificity and sensitivity of our algorithm is maximized across all mutation datasets

The Capritotti and Altman (2011) benchmark comprises three mutation datasets: the cancer and neutral only (CNO) mutation dataset assesses the performance of computational prediction algorithms when tasked with discriminating between driver mutations and neutral (germ line) polymorphisms; the cancer, neutral and other disease (CND) mutation dataset is used to evaluate the performance of computational prediction algorithms when tasked with distinguishing between cancer-associated and other germ line mutations (both disease-causing and neutral polymorphisms); and the synthetic mutation dataset measures the performance of computational prediction algorithms when differentiating between somatic driver and passenger mutations. Therefore, to derive a prediction threshold capable of being applied under all conditions, we plotted the distribution of the predicted magnitude of effect for all mutations in the Capriotti and Altman benchmark using a leave-one-out cross-validation procedure (Fig. 1). From this, we calculated a prediction threshold at which the specificity and sensitivity of our algorithm were both maximized across the mutation datasets: −0.75. Using this threshold, we observed that a large proportion of driver mutations (92%) fell below our prediction threshold, whereas the vast majority of germ line polymorphisms (disease-causing/putative neutral mutations) and passenger mutations fell above our prediction threshold, 94 and 87%, respectively.

### 3.2 An independent benchmark against other computational prediction algorithms

Using the Capriotti and Altman (2011) mutation datasets, we performed an independent benchmark comparing the performance of our method with the performance of two generic computational prediction algorithms: SIFT (Ng and Henikoff, 2001) and PolyPhen-2 (Adzhubei *et al.*, 2010); alongside two cancer-specific computational prediction algorithms: Mutation Assessor (Reva *et al.*, 2011) and SPF-Cancer (Capriotti and Altman, 2011). For this analysis, we obtained SIFT and PolyPhen-2 predictions using the corresponding algorithms’ batch submission facilities, whereas Mutation Assessor predictions were collected using the available web service, and SPF-Cancer predictions were provided by the corresponding author on request (as no batch submission is available). The algorithm’s default parameters and prediction thresholds were applied throughout our analysis.

**Fig. 2.**
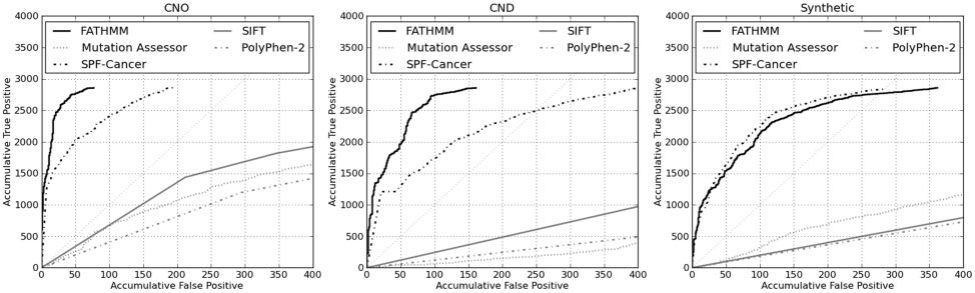
ROC curves showing the cumulative true positive rate versus the cumulative false positive rate for the computational prediction algorithms evaluated in our independent benchmark

First, using the cancer and neutral only (CNO) mutation dataset, we assessed the performance of these algorithms when tasked with distinguishing between driver mutations and putatively neutral polymorphisms. In addition, using the cancer, neutral and other disease (CND) mutation dataset, we assessed the performance of these algorithms when tasked with differentiating between driver mutations and other disease-causing mutations (non-neoplasm). From Table 2, and in terms of performance accuracies, it would seem that our method is the best-performing algorithm across these mutation datasets (94 and 93%, respectively). Using the synthetic mutation dataset, we assessed the performance of these algorithms when tasked with discriminating between somatic driver and passenger mutations. Here, our method outperforms SIFT, PolyPhen-2 and Mutation Assessor; it is comparable with SPF-Cancer (89 and 90%, respectively). Next, we compared the performance of our domain-based algorithm with the performance of our novel extension (capturing regions falling outside of conserved protein domains). We observed similar performances both within and outside conserved protein domains and concluded that our extension (and the corresponding weighting scheme) was just as effective as our domain-based algorithm when predicting the functional consequences of cancer-associated mutations (Supplementary Table S2). Finally, we plotted receiver operating characteristic (ROC) curves in the form of cumulative true positive/false positive plots centred on a conservative 1% error rate (Fig. 2). These curves re-affirm the comparable performances between our algorithm and SPF-Cancer. In addition, these curves demonstrate the relatively poor performances of ‘generic’ computational prediction algorithms, such as SIFT and PolyPhen-2, when applied to predict the functional consequences of cancer-associated mutations.

**Table 2.** Performance of computational prediction methods using the Capriotti and Altman benchmarking datasets

Method	tp	fp	tn	fn	Accuracy^a^	Precision^a^	Specificity^a^	Sensitivity^a^	NPV^a^	MCC^a^
Cancer and neutral only (CNO)										
SIFT	2180	560	1266	982	0.69	0.69	0.69	0.69	0.69	0.38
PolyPhen-2^b^	2421	1244	1894	656	0.70	0.66	0.60	0.79	0.74	0.40
Mutation Assessor	2403	1004	2155	751	0.72	0.71	0.68	0.76	0.74	0.45
SPF-Cancer	2876	196	2967	287	0.92	0.94	0.94	0.91	0.91	0.85
FATHMM	2858	77	3077	300	**0.94**	**0.97**	**0.98**	**0.91**	**0.91**	**0.88**
Cancer, neutral and other disease (CND)										
SIFT	2180	943	745	982	0.57	0.55	0.44	0.69	0.59	0.14
PolyPhen-2^b^	2421	1921	1238	656	0.56	0.54	0.34	0.79	0.62	0.14
Mutation Assessor	2403	1921	1238	751	0.58	0.56	0.39	0.76	0.62	0.17
SPF-Cancer	2876	418	2745	287	0.89	0.87	0.87	0.91	0.91	0.78
FATHMM	2858	161	2933	300	**0.93**	**0.95**	**0.95**	**0.91**	**0.91**	**0.85**
Synthetic										
SIFT	2180	1431	1434	982	0.59	0.58	0.50	0.69	0.62	0.19
PolyPhen-2^b^	2421	1902	985	656	0.56	0.54	0.34	0.79	0.62	0.14
Mutation Assessor	2403	1474	1432	751	0.63	0.60	0.49	0.76	0.67	0.26
SPF-Cancer	2859	297	2866	304	**0.90**	**0.91**	**0.91**	0.90	0.90	**0.81**
FATHMM	2858	362	2710	300	0.89	0.88	0.88	**0.91**	**0.90**	0.79

*Note:* tp, fp, tn, fn refer to the number of true positives, false positives, true negatives and false negatives, respectively. Bold values indicate the best performing method across the corresponding performance statistics. ^a^Accuracy, precision, specificity, sensitivity, NPV and MCC are calculated ‘from normalized numbers. ^b^‘Possibly damaging’ predictions are classified as pathogenic.

As our prediction threshold was derived using the same mutation datasets used in this benchmark (albeit using a leave-one-out analysis), and a large proportion of driver mutations is also present in our training data, we recognize the potential for bias in the observed performances. Therefore, to alleviate this bias, we further performed a 20-fold cross-validation procedure (Supplementary Table S3). We observed no significant deviations in the performance measures reported earlier in the text and, therefore, concluded that the performance of our algorithm is not an artefact of our weighting scheme.

Finally, to enable a direct (and fair) comparison between our algorithm and another leading computational prediction algorithm, CHASM (Carter *et al.*, 2009), we performed the same 2-fold cross-validation procedure used in (Capriotti and Altman, 2011) using the synthetic dataset. Here, we observed an improved performance when using our algorithm (Table 3). Furthermore, we observed no significant deviations from our original performance measures reported earlier in the text.

**Table 3.** A performance comparison using a 2-fold cross-validation procedure

Method	Accuracy	Precision	Specificity	Sensitivity	NPV	MCC
CHASM	0.80	0.85	0.87	0.73	0.76	0.60
FATHMM	**0.87**	**0.88**	**0.88**	**0.86**	**0.86**	**0.74**

*Note:* The performances of CHASM have been reproduced with permission from Capriotti and Altman (2011), Copyright 2013, Elsevier. Bold values indicate the best performing method across the corresponding performance statistics.

**Table 4.** Performance of computational prediction methods using the Gonzalez-Perez *et al.* benchmarking datasets

Dataset	SIFT		PolyPhen-2		Mutation assessor		TransFIC		FATHMM		
	Acc.	MCC	Acc.	MCC	Acc.	MCC	Acc.	MCC	Acc.	MCC	Threshold
COSMIC 2 + 1	0.49	0.10	0.59	0.06	0.30	0.80	**0.93**	0.50	**0.93**	**0.63**	−3.50
COSMIC 5 + 1	0.49	0.12	0.60	0.09	0.32	0.90	**0.97**	**0.57**	0.95	**0.57**	−3.50
COSMIC 2/POL	0.70	0.32	0.79	0.39	0.80	0.91	**0.93**	**0.86**	**0.93**	0.84	−1.50
COSMIC 5/POL	0.71	0.32	0.86	0.41	0.71	0.96	**0.98**	0.76	0.97	**0.89**	−1.50
COSMIC D/O	0.48	0.09	0.61	0.10	0.18	0.78	0.88	0.25	**0.90**	**0.35**	−3.00
COSMIC D/POL	0.70	0.29	0.85	0.42	0.64	0.92	0.94	0.69	**0.95**	**0.86**	−0.75
COSMIC CGC/NONCGC	0.44	0.08	0.56	0.07	0.16	0.78	0.85	0.50	**0.91**	**0.55**	−1.60
WG 2/1	0.84	0.02	0.71	0.01	0.10	0.89	0.96	0.23	**0.97**	**0.31**	−3.50
WG CGC/NONCGC	0.42	0.11	0.56	0.11	0.34	0.90	0.94	**0.52**	**0.95**	0.39	−2.80

*Note:* The performances of alternative computational prediction algorithms have been reproduced with permission from Gonzalez-Perez *et al.* (2012; Open Access Article). Bold values indicate the best performing method across the corresponding benchmark.

### 3.3 A performance comparison with a published review

In addition to performing our own benchmark, we downloaded and used the Gonzalez-Perez *et al.* (2012) benchmark (comprising nine mutation datasets) to compare the performance of our algorithm with four alternative computational prediction algorithms: SIFT (Ng and Henikoff, 2001), PolyPhen-2 (Adzhubei *et al.*, 2010), Mutation Assessor (Reva *et al.*, 2011) and TransFIC (Gonzalez-Perez *et al.*, 2012). For this analysis, we opted to compare our algorithm with the Mutation Assessor TransFIC, as it has been shown to outperform the SIFT TransFIC and PolyPhen-2 TransFIC. In accordance with (Gonzalez-Perez *et al.*, 2012), and to enable a fair comparison to be made between our algorithm and the Mutation Assessor TransFIC, we adjusted our prediction thresholds across the nine mutation datasets to maximize the Matthews correlation coefficient (MCC) of our algorithm. Here, our algorithm outperforms SIFT, PolyPhen-2 and Mutation Assessor across all mutation datasets. In addition, it seems our algorithm is comparable with the Mutation Assessor TransFIC (Table 4). The performance of our algorithm using our standard prediction threshold is documented in Supplementary Table S4.

### 3.4 Benefits of a disease-specific weighting scheme

To better understand the potential benefits of incorporating a cancer-specific weighting scheme into our algorithm, we compared the score/prediction assignments for all mutations in the Capriotti and Altman (2011) benchmark using a cancer-specific weighting scheme with the score/prediction assignments for the same mutations using our original inherited-disease weighting scheme. As expected, the odds of identifying driver and passenger mutations were 7.92 (CI: 6.82, 9.22) and 1.95 (CI: 1.69, 2.25) times greater, respectively, when using a cancer-specific weighting scheme. Furthermore, the odds of correctly identifying other disease-causing mutations as having no effect on tumour progression were 75.48 (CI: 59.70, 96.17) times greater when using a cancer-specific weighting scheme. The observed performance gain illustrates the ability of our algorithm to not only distinguish between driver and passenger mutations but also to discriminate between cancer-associated mutations and other germ line mutations (both disease-associated and neutral polymorphisms).

## 4 DISCUSSION

In this article, we described an adaptation to the Functional Analysis through Hidden Markov Models (FATHMM) algorithm (Shihab *et al.*, 2013) in which a cancer-specific weighting scheme was incorporated to potentiate the functional analysis of driver mutations. The performance of our method was then benchmarked against four alternative computational prediction algorithms: SIFT (Ng and Henikoff, 2001) and PolyPhen-2 (Adzhubei *et al.*, 2010), Mutation Assessor (Reva *et al.*, 2011) and SPF-Cancer (Capriotti and Altman, 2011); using the Capriotti and Altman (2011) benchmarking datasets. In terms of performance accuracies, FATHMM seems to be the best performing method available when assigned with the task of distinguishing between driver mutations and other germ line polymorphisms (both disease-causing and neutral). Furthermore, when tasked with discriminating between driver and passenger mutations (somatic), our method seems to perform as well as the alternative leading prediction algorithm: SPF-Cancer. Although the performance of our algorithm in this category does not represent an improvement over SPF-Cancer, our method offers a large-scale/high-throughput batch submission facility capable of analysing all foreseeable genomic/cancer datasets—an important facility that is not offered with SPF-Cancer. In addition, to facilitate a comparison between our algorithm and another leading computational prediction algorithm: CHASM (Carter *et al.*, 2009), we performed a 2-fold cross-validation procedure and observed an improved performance when using our method. We also compared the performance of our algorithm with four computational prediction algorithms: SIFT (Ng and Henikoff, 2001), PolyPhen-2 (Adzhubei *et al.*, 2010), Mutation Assessor (Reva *et al.*, 2011) and TransFIC (Gonzalez-Perez *et al.*, 2012), using a published benchmark (Gonzalez-Perez *et al.*, 2012). Once again, we observed improved performance accuracies over traditional computational prediction algorithms: SIFT, PolyPhen-2 and Mutation Assessor; and we noted comparable performances with the Mutation Assessor TransFIC.

In any fair comparison, care should be taken to reduce the potential overlap between the mutation datasets used for training and testing; however, this level of testing is not possible, as it would require obtaining and retraining each algorithm with common datasets. To remove the potential bias in our results, we performed a 20-fold cross-validation procedure across our benchmark. From this analysis, we observed no significant deviations in the performance of our algorithm and, therefore, concluded that the performances observed were not an artefact of the weighting scheme used.

The potential benefits of incorporating cancer-specific information into our predictions were assessed by comparing the performance of our cancer-specific weighting scheme with the performance of our original inherited-disease weighting scheme. In accordance with previous findings (Kaminker *et al.*, 2007a), we observed some similarities in driver scores/predictions between the two weighting schemes. However, we noted improved odds in identifying driver/passenger mutations using a cancer-specific weighting scheme. Unsurprisingly, we also noted significantly improved odds in correctly classifying disease-causing (non-neoplasm) mutations as having no effect on tumour progression. Therefore, by incorporating a cancer-specific weighting scheme, we have shown that our method is capable of identifying mutations that directly contribute to carcinogenesis, irrespective of other underlying disease associations.

To facilitate the analysis of large-scale cancer genomic datasets, our public web server (available at http://fathmm.biocompute.org.uk) provides unrestricted and near instant predictions for all possible amino acid substitutions within the human proteome. For example, we were capable of annotating the entire COSMIC (Bamford *et al.*, 2004) database—comprising of over half a million mutations—in <1 h using a single processing core. In addition, we also provide an open-source software package allowing users to run our algorithm using their high-performance computing systems.

## Supplementary Material

Supplementary Data
