# Supplementary material for: Predicting the functional consequences of cancer-associated amino acid substitutions
Source: Bioinformatics. 2013 May 17;29(12):1504–10. doi: 10.1093/bioinformatics/btt182 (PMC3673218; doi:10.1093/bioinformatics/btt182)
Supplement: Supplementary Data [file supp_29_12_1504__index.html]

Predicting the functional consequences of cancer-associated amino acid substitutions — Predicting the functional consequences of cancer-associated amino acid substitutions — Supplementary Data 

# Predicting the functional consequences of cancer-associated amino acid substitutions

## Supplementary Data

files

**Files in this Data Supplement:**

- Supplementary Data - doc file
