# Supplementary material for: Predicting the functional consequences of cancer-associated amino acid substitutions
Source: Bioinformatics. 2013 May 17;29(12):1504–10. doi: 10.1093/bioinformatics/btt182 (PMC3673218; doi:10.1093/bioinformatics/btt182)
Supplement: Supplementary Data [file supp_btt182_Supp_Info_Revised_19March2013.doc]

**SUPPLEMENTARY METHODS**

**Deriving a Domain-Based Weighting Scheme**


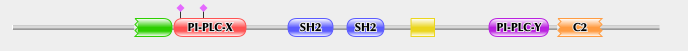


Our domain-based pathogenicity weights are derived as follows: first, the full length protein sequence(s) for all mutations in our training data (CanProVar and UniProt) are annotated with conserved protein domains using the SUPERFAMILY and Pfam database. Next, we calculate the relative frequencies of cancer-associated and putative neutral polymorphisms falling within these regions. For example, in the above, we observe two cancer-associated mutations falling within the PI-PLC-X domain. Assuming no other mutation(s) within our training dataset falls within the corresponding model, our pathogenicity weights *W_c_* and *W_p_* are initialized as 2 and 0, respectively.

**Extending our Weighting Scheme outside Conserved Protein Domains**

Following a similar procedure to the above, homologous sequences from the SwissProt/TrEMBL database are collected and aligned using the *JackHMMER* algorithm (one iteration with the --*hand* and --*domtblout* parameters applied). The --*domtblout* parameter produces a report describing regions of similarity between our query sequence and the homologous sequences identified. These regions are interrogated and our pathogenicity weights, *W_c_* and *W_p_*, are incremented according to the relative frequencies of cancer-associated and putative neutral polymorphisms falling within these regions.

**SUPPLEMENTARY TABLES**

**Supp. Table 1.** Overlap Between our Training and Benchmarking Datasets

|  | **# of Positives** | **% Overlap** | **# of Negatives** | **% Overlap** |
| --- | --- | --- | --- | --- |
| ***Capriotti And Altman (2011)*** | | | | |
| CNO | 3,163 | 84% | 3,163 | 45% |
| CND | 3,163 | 84% | 3,163 | 23% |
| Synthetic | 3,163 | 84% | 3,163 | < 1% |
|  |  |  |  |  |
| ***Gonzalez-Perez e.t al.(2012)*** | | | | |
| COSMIC 2+1 | 3,978 | 30% | 39,850 | 8% |
| COSMIC 5+1 | 1,631 | 31% | 39,850 | 8% |
| COSMIC 2/POL | 3,978 | 30% | 8,040 | 82% |
| COSMIC 5/POL | 1,631 | 31% | 8,040 | 82% |
| COSMIC D/O | 2,151 | 77% | 41,664 | 6% |
| COSMIC D/POL | 2,151 | 77% | 8,040 | 82% |
| COSMIC CGC/NONCGC | 4,865 | < 1% | 34,827 | 5% |
| WG 2/1 | 790 | < 1% | 24,079 | < 1% |
| WG CGC/NONCGC | 1,302 | < 1% | 22,983 | < 1% |

**Supp. Table 2.** Performance of our Algorithm Both Within and Outside Conserved Protein Domains

|  | **tp** | **fp** | **tn** | **fn** | **Accuracy**^†^ | **Precision**^†^ | **Specificity**^†^ | **Sensitivity**^†^ | **NPV**^†^ | **MCC**^†^ |
| --- | --- | --- | --- | --- | --- | --- | --- | --- | --- | --- |
| ***Cancer and Neutral Only (CNO)*** | | | | | | | | | | |
| Within Domains | 2,372 | 47 | 1,809 | 254 | 0.94 | 0.97 | 0.97 | 0.90 | 0.91 | 0.88 |
| Outside Domains | 486 | 30 | 1,268 | 46 | 0.95 | 0.98 | 0.98 | 0.91 | 0.92 | 0.89 |
|  |  |  |  |  |  |  |  |  |  |  |
| ***Cancer, Neutral and other Disease (CND)*** | | | | | | | | | | |
| Within Domains | 2,372 | 140 | 2,110 | 254 | 0.92 | 0.94 | 0.94 | 0.90 | 0.91 | 0.84 |
| Outside Domains | 486 | 21 | 823 | 46 | 0.94 | 0.97 | 0.98 | 0.91 | 0.92 | 0.89 |
|  |  |  |  |  |  |  |  |  |  |  |
| ***Synthetic*** | | | | | | | | | | |
| Within Domains | 2,372 | 249 | 1,683 | 254 | 0.89 | 0.88 | 0.87 | 0.90 | 0.90 | 0.77 |
| Outside Domains | 486 | 113 | 1,027 | 46 | 0.91 | 0.90 | 0.90 | 0.91 | 0.91 | 0.81 |

*tp*, *fp*, *tn*, *fn* refer to the number of true positives, false positives, true negatives and false negatives, respectively.

^†^ *Accuracy, Precision, Specificity, Sensitivity, NPV* and *MCC* are calculated from normalised numbers

**Supp. Table 3.** Performance of our Algorithm using a 20-Fold Cross-Validation Procedure across the Capriotti and Altman Benchmarking Datasets

|  | **Accuracy**^†^ | **Precision**^†^ | **Specificity**^†^ | **Sensitivity**^†^ | **NPV**^†^ | **MCC**^†^ |
| --- | --- | --- | --- | --- | --- | --- |
| CNO | 0.93 | 0.97 | 0.97 | 0.90 | 0.90 | 0.87 |
| CND | 0.92 | 0.94 | 0.95 | 0.90 | 0.90 | 0.85 |
| Synthetic | 0.89 | 0.88 | 0.88 | 0.90 | 0.90 | 0.78 |

^†^ *Accuracy, Precision, Specificity, Sensitivity, NPV* and *MCC* are calculated from normalised numbers

**Table 4.** Performance of our Algorithm using the Default Threshold across the Gonzalez-Perez et. al. Benchmarking Datasets

|  | **Accuracy** | **MCC** |
| --- | --- | --- |
| COSMIC 2+1 | 0.83 | 0.47 |
| COSMIC 5+1 | 0.83 | 0.37 |
| COSMIC 2/POL | 0.92 | 0.82 |
| COSMIC 5/POL | 0.95 | 0.84 |
| COSMIC D/O | 0.80 | 0.37 |
| COSMIC D/POL | 0.95 | 0.86 |
| COSMIC CGC/NONCGC | 0.87 | 0.50 |
| WG 2/1 | 0.88 | 0.13 |
| WG CGC/NONCGC | 0.89 | 0.29 |
